# Plasma gelsolin promotes re-epithelialization

**DOI:** 10.1038/s41598-018-31441-2

**Published:** 2018-09-03

**Authors:** J. Wittmann, J. Dieckow, H. Schröder, U. Hampel, F. Garreis, C. Jacobi, A. Milczarek, K. L. Hsieh, B. Pulli, J. W. Chen, S. Hoogeboom, L. Bräuer, F. P. Paulsen, S. Schob, M. Schicht

**Affiliations:** 1 Department of Functional and Clinical Anatomy, Friedrich-Alexander-University Erlangen-Nürnberg, Erlangen, Germany; 2 Department of Ophthalmology, University of Leipzig, Leipzig, Germany; 3 Schepens Eye Research Institute and Massachusetts Eye and Ear, Harvard Medical School, Boston, MA USA; 4 Department of Ophthalmology, University Medical Center, Johannes Gutenberg University, Mainz, Germany; 5 Clinic of Ophthalmology, Friedrich-Alexander-University Erlangen-Nürnberg, Erlangen, Germany; 6 Department of Medical Imaging, Taipei Medical University Hospital, Taipei, Taiwan; 7 Research Center of Translational Imaging, College of Medicine, Taipei Medical University, Taipei, Taiwan; 8 Center for Systems Biology and Institute for Innovation in Imaging, Massachusetts General Hospital and Harvard Medical School, Boston, MA USA; 9 Frauenhofer Institute for Supply Chain Services SCS, Nürnberg, Germany; 10 Department of Neuroradiology, University of Leipzig, Leipzig, Germany

## Abstract

Woundhealing disorders characterized by impaired or delayed re-epithelialization are a serious medical problem that is painful and difficult to treat. Gelsolin (GSN), a known actin modulator, supports epithelial cell regeneration and apoptosis. The aim of this study was to estimate the potential of recombinant gelsolin (rhu-pGSN) for ocular surface regeneration to establish a novel therapy for delayed or complicated wound healing. We analyzed the influence of gelsolin on cell proliferation and wound healing *in vitro*, *in vivo*/*ex vivo* and by gene knockdown. Gelsolin is expressed in all tested tissues of the ocular system as shown by molecular analysis. The concentration of GSN is significantly increased in tear fluid samples of patients with dry eye disease. rhu-pGSN induces cell proliferation and faster wound healing *in vitro* as well as *in vivo/ex vivo*. TGF-β dependent transcription of SMA is significantly decreased after GSN gene knockdown. Gelsolin is an inherent protein of the ocular system and is secreted into the tear fluid. Our results show a positive effect on corneal cell proliferation and wound healing. Furthermore, GSN regulates the synthesis of SMA in myofibroblasts, which establishes GSN as a key protein of TGF-β dependent cell differentiation.

## Introduction

Healthy cornea is a transparent avascular tissue and is formed by three cellular layers (epithelium, stroma, endothelium) distinctively separated by acellular membranes (Bowman’s and Descemet’s membrane). The most superficial cell layer to which the tear film attaches is the corneal epithelium.

Corneal surface diseases such as injuries, dry eye disease (DED), systemic inflammatory disorders with ocular involvement (e.g. Stevens-Johnson syndrome, ocular cicatricial pemphigoid), chemical or thermal burns, and in some cases also medical or surgical interventions, may be accompanied by impaired or delayed re-epithelialization of the corneal epithelium and can result in deterioration of visual acuity^1^. Such impaired wound healing can also occur in other epithelia, for example various mucosal and dermal tissues, and is observed with increasing frequency, especially in the elderly and in patients suffering from chronic conditions like diabetes mellitus or metabolic syndrome^2^. Delayed epithelial closure may result in extremely painful chronic wounds or even (unilateral) loss of vision. It may also be a consequence of infections due to impaired epithelial barrier function. In more severe injuries that go beyond epithelial damage, the activation of proliferative fibroblasts or myofibroblasts can be responsible for the development of contractile scar tissue^3^. Subsequent opaqueness and haze can severely compromise visual acuity. According to a recent WHO study, up to 55 million injuries involving the ophthalmic system occur worldwide every year^4,5^. The most frequent causes of vision loss due to corneal conditions are traumata (related to accidents, occupational mishaps and sports activities), corneal ulceration or, increasingly, surgical interventions such as radial keratotomy, photorefractive keratectomy or laser-*in-situ*-keratomileusis (LASIK) to correct refractive errors^6^.

Wound healing is a complex response to injuries of tissues and cell clusters. The main steps in wound healing are inflammation, tissue formation and tissue remodeling^7–9^. Injuries activate inflammatory cells, trigger release of mediators and cause contraction of blood vessels and formation of blood platelet clots. Furthermore, migration of keratinocytes, angiogenesis and, ultimately, wound closure succeeded by tissue remodeling are part of the wound healing cascade^8,10–12^.

A main part of wound healing is a complex interaction between macrophages, granulocytes and fibroblasts^10^. A key factor in the underlying signaling cascades is transforming growth factor-β (TGF-β). TGF-β activates epithelial to mesenchymal conversion, which is important for sufficient wound healing, and also regulates maturation from fibroblasts to myofibroblasts, which contribute to wound contraction^13,14^. The decisive role of myofibroblasts in the process of wound healing is discussed controversially, but the prevailing opinion is that they are necessary^15,16^. TGF-β is an important regulator of epithelial wound healing and leads to an increased gene expression of gelsolin (GSN). GSN, on the other hand, can modulate TGF-β-induced cell differentiation^17^.

GSN is a multifunctional actin-binding protein that is produced by almost every cell type^18^. The GSN gene undergoes post-transcriptional splicing, yielding a cytoplasmatic isoform (cytoplasmatic gelsolin - cGSN) and a secreted plasmatic isoform (plasmatic gelsolin - pGSN) [20]. cGSN is essential to the processes of cellular locomotion and morphogenesis, as it regulates assembly and degradation of intracellular actin filaments by capping, nucleation and severing^19^. It is therefore of special importance for phagocytosis and the proper function of thrombocytes^20,21^.

pGSN,on the other hand, is produced mainly by skeletal muscle cells and distributed into the blood^22^. pGSN binds (scavenges) and removes free toxic actin after cell damage through non-proteolytic deterioration. Circulating pGSN seems to be a humoral reserve recruited in case of inflammation or trauma. Other functions of pGSN are elimination of bacterial toxins, e.g. lipopolysaccharide and lipoteichoic acid, and amelioration of immune response by reducing the release of proinflammatory interleukins^18,23–26^. Witke *et al*. showed that skin fibroblasts of GSN null mice demonstrated severe impairments in cell motility based on an incapacity to reorganize their actin skeleton^27^.As such, GSN has tremendous potential to enhance wound repair and might also qualify as a future therapeutic.

## Results

### GSN is expressed and synthesized in human and murine ocular tissues and cell lines

**Figure 1:**
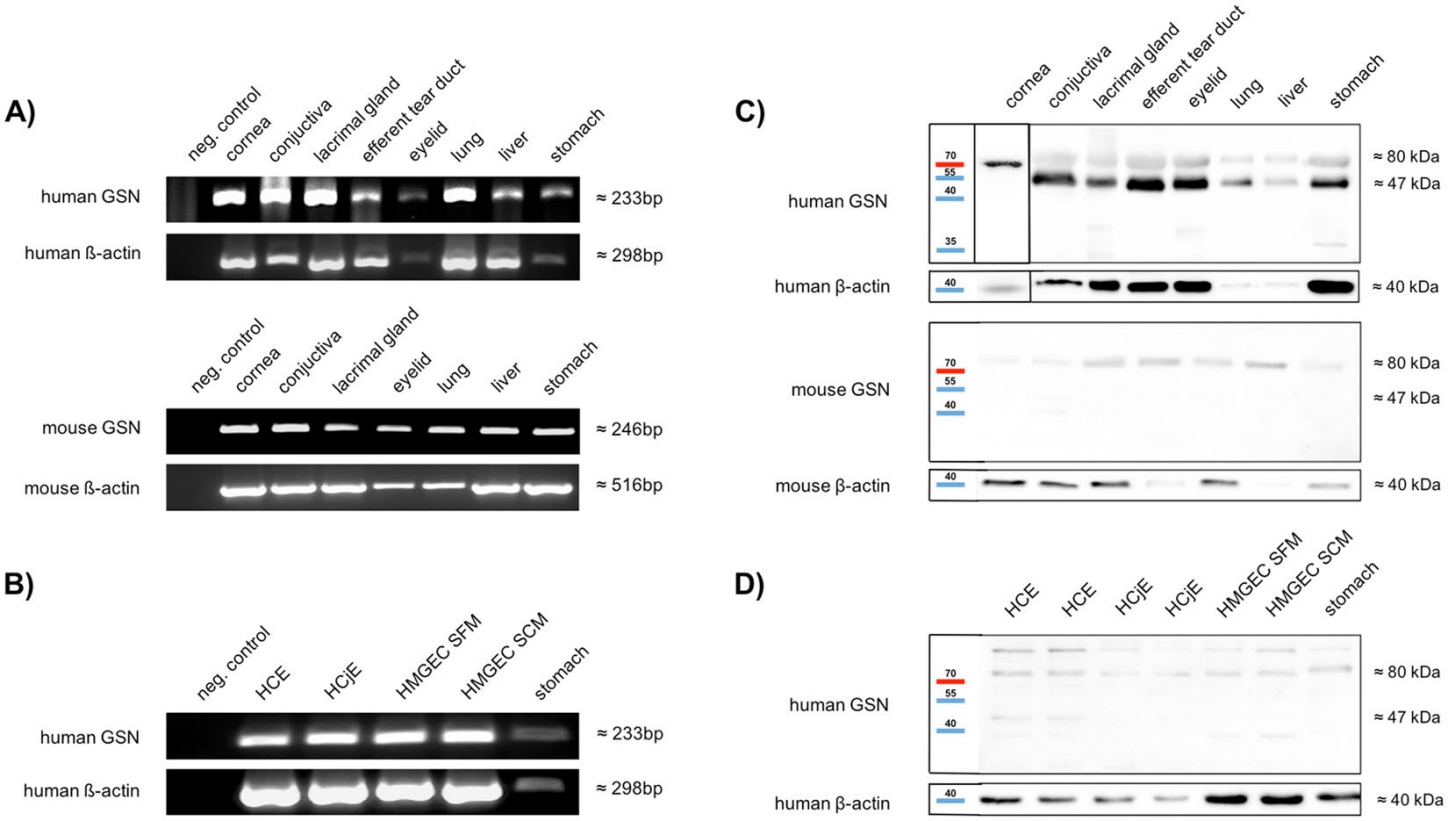
RT-PCR and Western blot analysis of GSN expression. (**A**,**B**) Expression of specific GSN mRNA amplification products in human (cornea (n = 3), conjunctiva (n = 3), lacrimal gland (n = 3), efferent tear duct (n = 3), eyelid (n = 3), lung (n = 3), liver (n = 1) and stomach (n = 1)) and mouse tissues (cornea (n = 6), conjunctiva (n = 6), lacrimal gland (n = 6), eyelid (n = 6), lung (n = 5), liver (n = 6) and stomach (n = 5)) and three different human cell lines (i.e. HCE = human cornea epithelial cells (n = 3); HCjE = human conjunctiva epithelial cells (n = 3); HMGEC SFM = human meibomian gland epithelial cells cultured with serum-free medium (n = 3); HMGEC SCM = human meibomian gland epithelial cells cultured with serum containing medium to induce differentiation (n = 3). All negative controls without template cDNA. Stomach, liver and lung served as positive controls. (**C**,**D**) Western blot analysis of human (cornea (n = 3), conjunctiva (n = 3), lacrimal gland (n = 3), efferent tear duct (n = 3), eyelid (n = 3), lung (n = 3), liver (n = 1) and stomach (n = 1)) and mouse tissue (cornea (n = 6), conjunctiva (n = 6), lacrimal gland (n = 6), eyelid (n = 6), lung (n = 5), liver (n = 6) and stomach (n = 5)) samples and three different human cell lines (HCE = human cornea epithelial cells (n = 3); HCjE = human conjunctiva epithelial cells (n = 3); HMGEC SFM = human meibomian gland epithelial cells cultured with serum free medium (n = 3); HMGEC SCM = human meibomian gland epithelial cells cultured with serum containing medium to induce differentiation (n = 3)) using an anti-GSN antibody. Stomach, liver and lung tissue samples served as positive controls.

**Figure 2:**
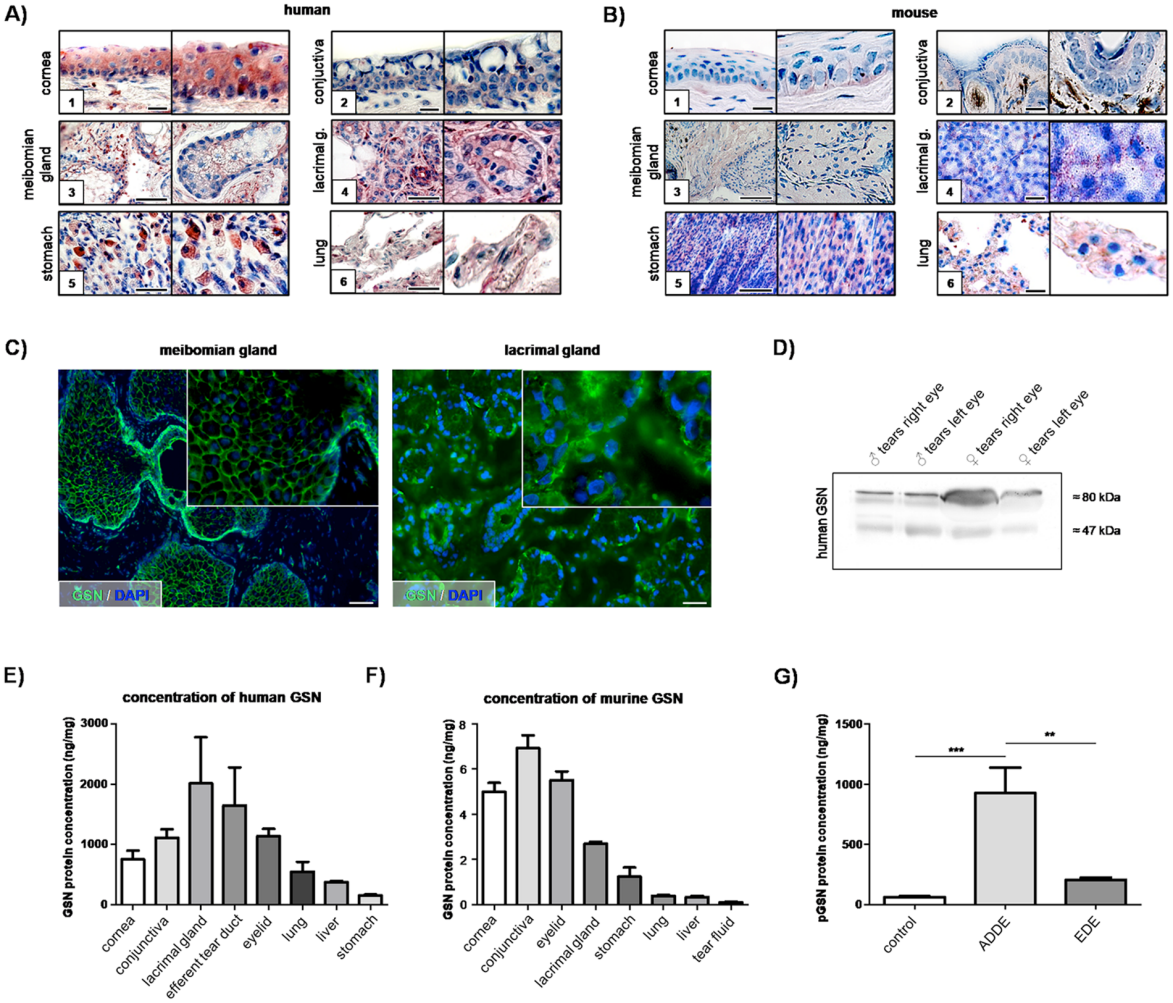
Localization and quantification of GSN in tissues of the ocular surface and lacrimal apparatus as well as in healthy tears and tears from patients suffering from aqueous-deficient and evaporative forms of dry eye disease. Immunohistochemical localization of GSN in human and murine cornea (**1A,2B**), conjunctiva (**2A,2B**) meibomian gland (**3A,3B**) lacrimal gland (**4A,4B**), stomach (**5A,5B**) and lung (**6A,6B**). Stomach and lung are used as positive controls. Red staining indicates positive reactivity of the antibody. The right part of each picture shows magnification. Scale bar: [1A],[2A],[1B],[2B],[4B],[6B] 20 µm, [3A]–[6A] 50 µm, [3B],[5B] 100 µm. (**C**) Immunofluorescence detection of GSN in a human meibomian gland and a human lacrimal gland. (GSN: green; DAPI (4′,6-diamidino-2-phenylindole): blue). Scale bar: meibomian gland 200 µm, lacrimal gland 100 µm. (**D**) Western blot analysis of human tear fluid of healthy male and a female donors (each n = 1) using an anti-GSN antibody. (**E**,**F**) Quantification of GSN protein by enzyme-linked immunosorbent assay (ELISA) of human (**E**) and mouse (**F**) tissue samples. (**E**) Quantification of GSN in human tissues ELISA. Amount of GSN is in relation to total protein amount. (**F**) Quantification of GSN in mouse tissue and tear fluid by ELISA. (**G**) Quantification of GSN in tear fluid by ELISA. Analyzed samples were from healthy (n = 10), aqueous-deficient dry eye (ADDE) (n = 14) and hyperevaporation dry eye (EDE) (n = 14). Statistical significance: **p ≤ 0.005, ***p ≤ 0.0005.

Tissue samples from human and murine cornea, conjunctiva, lacrimal gland, efferent tear duct, eyelid, lung, liver and stomach as well as three different human epithelial cell lines, i.e. a human corneal epithelial cell line (HCE), a human conjunctival epithelial cell line (HCjE) and a human meibomian gland epithelial cell line (HMGEC) were tested positive for GSN mRNA (Fig. 1A,B). ß-actin served as loading control for all samples. The sequenced PCR bands were in accordance with the expected sequences from GSN in the gene database (www.ncbi.com).Protein extracts from cornea, conjunctiva, lacrimal gland, efferent tear duct, eyelid, lung, liver, stomach, HCE, HCjE, HMGEC (Fig. 1C,D)and human tear fluid samples (Fig. 2D) were tested for presence of GSN by Western blot analysis. Specific GSN protein bands were detected at 80 kDa and 47 kDa, respectively.

For immunohistochemistry, paraffin-embedded tissue sections from cornea, conjunctiva, eyelid, lacrimal gland, stomach and lung were analyzed. All investigated tissue samples showed positive anti-GSN antibody reactivity (Fig. 2A,B). Negative control sections were negative for all tissues (data not shown).

**Human (Hu) cornea**: GSN was detected cytoplasmically in all epithelial cell layers of the corneal epithelium (Fig. 2A1). **Mouse (Mo) cornea**: GSN was only detected in the basal epithelial cell layer of the corneal epithelium (Fig. 2B1). **Hu-conjunctiva**: Epithelial cells of the conjunctiva revealed intracytoplasmatic reactivity (red staining) with the anti-GSN antibody, with the exception of the secretion product of intraepithelial goblet cells (Fig. 2A2). **Mo-conjunctiva**: GSN was visible in cells of the conjunctival epithelium (Fig. 2B2). **Hu-meibomian gland**: Meibocytes reacted positive with the anti-GSN antibody as did the lining cells of the excretory duct system (Fig. 2A3). **Mo-meibomian gland**: Murine meibocytes reacted positive with the antibody to GSN as well (Fig. 2B3). **Hu-lacrimal gland**: GSN reactivity was detected intracytoplasmatically within acinar cells (Fig. 2A4). **Mo-lacrimal gland**: There was a GSN reactivity in acinar cells (Fig. 2B4). **Hu-stomach**: Only parietal cells revealed a positive antibody reactivity (Fig. 2A5). **Mo-stomach**: The antibody to GSN reacted positive in all cells of mouse gastric glands (Fig. 2B5). **Hu-lung and Mo-lung:** GSN reactivity was visible in type I and type II alveolar epithelial cells (Fig. 2A6,B6).

Immunofluorescence showed presence of GSN in human lacrimal and meibomian glands (Fig. 2C). Green fluorescence indicates positive antibody reactivity, visible in particular in lining cells of the excretory ducts.

ELISA of human tissue protein extracts showed mean values of total (cytoplasmic and plasmatic) GSN protein concentrations between 156 and 2012 ng/mg in cornea, conjunctiva, meibomian gland, lacrimal gland, liver, stomach and lung (Fig. 2E,F, Table S1). Mouse tissue contained lower protein concentrations, ranging between 0.09 and 6.93 ng/mg (Fig. 2E,F, Table S1).

All human and murine tissues of the ocular surface and lacrimal system showed higher concentrations of GSN than the respective positive control tissues of liver, stomach and lung.

### The pGSN concentration is significantly increased in tear fluid of patients suffering from aqueous-deficient or evaporative DED

Quantification of pGSN in tear fluid of patients suffering from aqueous-deficient dry eye (ADDE) or evaporative dry eye (EDE) revealed a significantly (p ≤ 0.0005) higher pGSN concentration compared to tears from healthy volunteers (Fig. 2G, Table S1).

### The influence of rhu-pGSN on cell proliferation

In order to mimic inflammatory conditions similar to bacterial infection or DED, we challenged rhu-pGSN stimulated HCE cells with the bacterial toxin LPS and the inflammatory cytokine TNFα.

**Figure 3:**
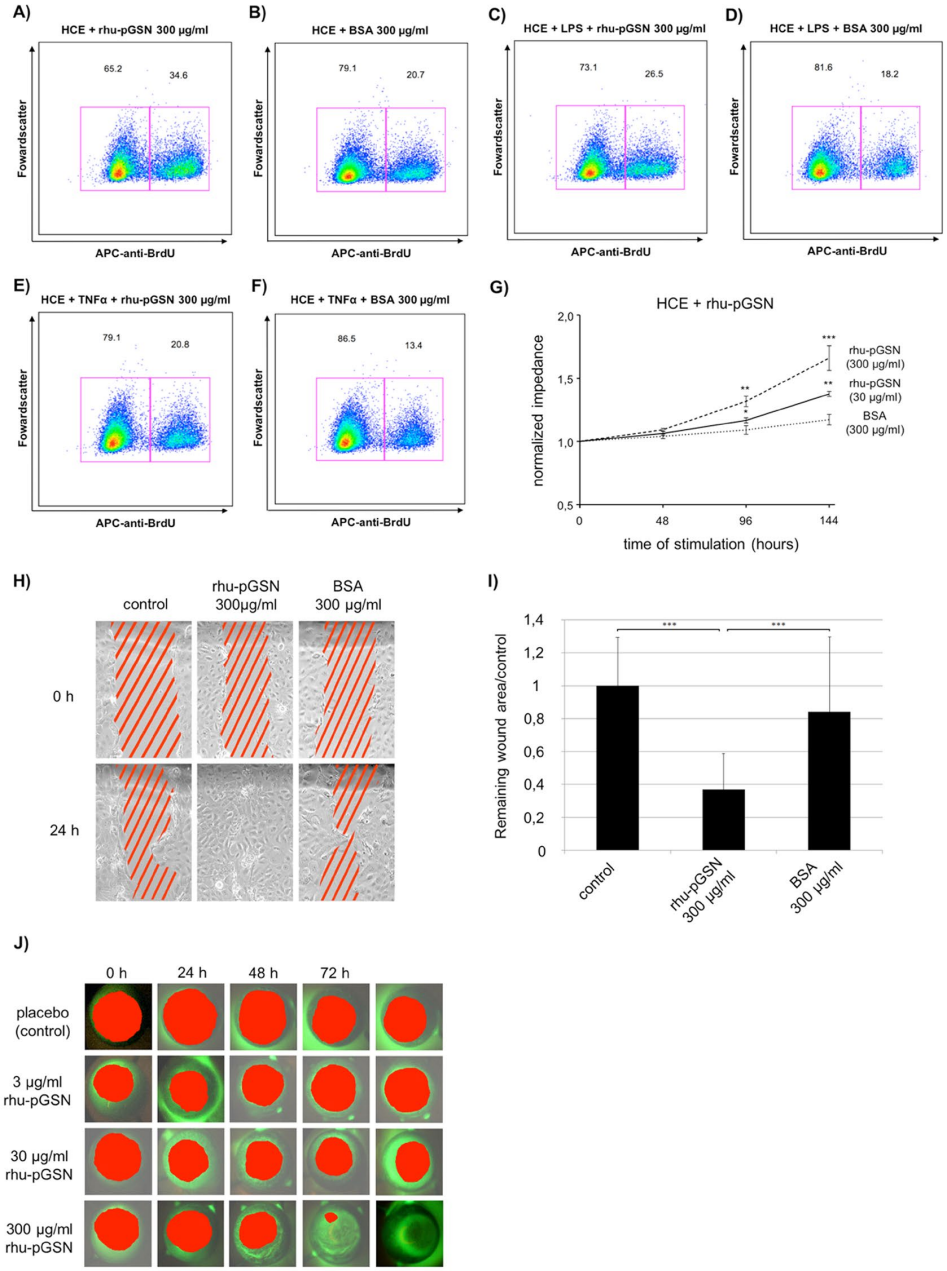
Proliferation and wound healing studies. (**A**–**F**) Cell proliferation of human corneal epithelial cells (HCE cell line) after treatment with recombinant human plasma gelsolin (rhu-pGSN) assessed with fluorescence-activated cell sorting (FACS). (**A**) Stimulation with rhu-pGSN and (**B**) devoid of rhu-pGSN (bovine serum albumin (BSA) protein control). (**C**,**E**) Stimulation with lipopolysaccharide (LPS) and tumor necrosis factor α (TNFα) in combination with rhu-pGSN. (**D**,**F**) Stimulation with LPS and TNFα in combination with BSA. (**G**) Normalized impedance after stimulation with rhu-pGSN assessed with Electric Cell-Substrate Impedance Sensing (ECIS®). Stimulations were BSA, 30 µg/ml rhu-pGSN, 300 µg/ml rhu-pGSN. Start of stimulation 0 hours. Statistical significance: *p≤0.05, **p≤0.005, ***p≤0.0005. (**H**) Scratch assay (n = 3) on HCE. Cells were wounded using a pippet tip. Wounded areas (red stripes) after 0 hours and 24 hours of incubation. (**I**) Restored wound area after scratch (n = 3) and 24 hour incubation with rhu-pGSN or BSA, compared to control values. The wound healing rates were significantly higher under stimulation with 300 µl/ml rhu-pGSN compared with no rhu-pGSN control as well as BSA protein control. Statistical significance: ***p < 0.001. (**J**) rhu-pGSN promotes re-epithelialization of corneal wounds in combined *in vivo*/*ex vivo* model. The measured wound areas are highlighted in red.

Flow cytometry analysis based on BrdU incorporation demonstrated increased HCE cell proliferation in cells treated with rhu-pGSN (Fig. 3A,B). Stimulation with 300 µg/ml rhu-pGSN yielded an increased cell proliferation rate (34.6%) compared to treatment with 300 µg/ml BSA (20.7%). LPS and TNFα were used to simulate a bacterial inflammation and an immune response, respectively. An additional treatment with 1 µg/ml lipopolysaccharides (LPS) or 10 ng tumor necrosis factor alpha (TNFα) had an expected diminishing effect on the proliferation rates (Fig. 3C–F). However, rates under rhu-pGSN stimulation were still markedly higher than under BSA control treatment (LPS: 26.5% versus 18.2%; TNFα: 20.8% versus 13.4%) (Table 1).

**Table 1:** Cell proliferation of human corneal epithelial cells (HCE cell line) after treatment with recombinant human plasma gelsolin (rhu-pGSN) assessed by fluorescence-activated cell sorting (FACS).

	Control	Rhup-GSN	Cell proliferation rate
without stimulant	20,7%	34,6%	↑
with LPS	18,2%	26,5%	↑
with TNF-α	13,4%	20,8%	↑

The arrow (↑) indicate an increase of the proliferation.

Quantification of the measured impedance or simplified as cell proliferation under rhu-pGSN stimulation was also analyzed by Electric Cell-Substrate Impedance Sensing (ECIS®) (Fig. 3G). Again, HCE cells were used as a model system for the ocular surface. There was a significant difference in cell proliferation rates between the control group (BSA) and both rhu-pGSN stimulated groups (30 µg/ml and 300 µg/ml) after 96 hours and 144 hours of stimulation. HCE cells treated with 300 µg/ml rhu-pGSN showed an even higher cell proliferation rate than cells treated with only 30 µg/ml rhu-pGSN (Fig. 3G).

### rhu-pGSN increases the wound closure rate *in vitro* and in a combined corneal *in vivo/ex vivo* defect model

The cell culture-based wound healing assay revealed increased gap closure of HCE cells in the presence of 300 μg/ml rhu-pGSN compared to no treatment or treatment with 300 µg/ml BSA (Fig. 3H,I). After 24 hours incubation time, the remaining wound area was significantly (p < 0.001) narrowed with a more than 2.5 fold increase in wound area restoration (Supplement Statistic PDF).

Positive effects of rhu-pGSN with regard to wound healing were also visible in a corneal wound healing mouse model (*in vivo/ex vivo* cornea defect model) (Fig. 3J). Red coloration indicates the wound surface. To compare the corneal wound healing process between placebo-treated and rhu-pGSN groups, we performed Kaplan-Meier analysis using digitized areas of the remaining corneal defect area over time. No significant reduction of the wounded area over time was observed in the untreated group as well as after treatment with 3 µg/ml rhu-pGSN. Treatment with30 µg/ml rhu-pGSN revealed a statistically not significant tendency to enhanced wound surface closure. However, mice corneae treated with 300 µg/ml rhu-pGSN showed complete *restitutio ad integrum* after 72 hours (Fig. 3J). These results were statistically significant (p = 0.0005) compared to the other treatment groups (Supplement Fig. S1).

### rhu-pGSN and rhuTGF-β promote expression of smooth muscle actin

To get a possible starting point in which way rhu-pGSN could interact in the cascade of wound healing we performed the experiments with rhuTGF-β and SMA. As mentioned in the introduction section TGF-β provides conversion of cells. Expression of SMA is a verification of cell conversion.

**Figure 4:**
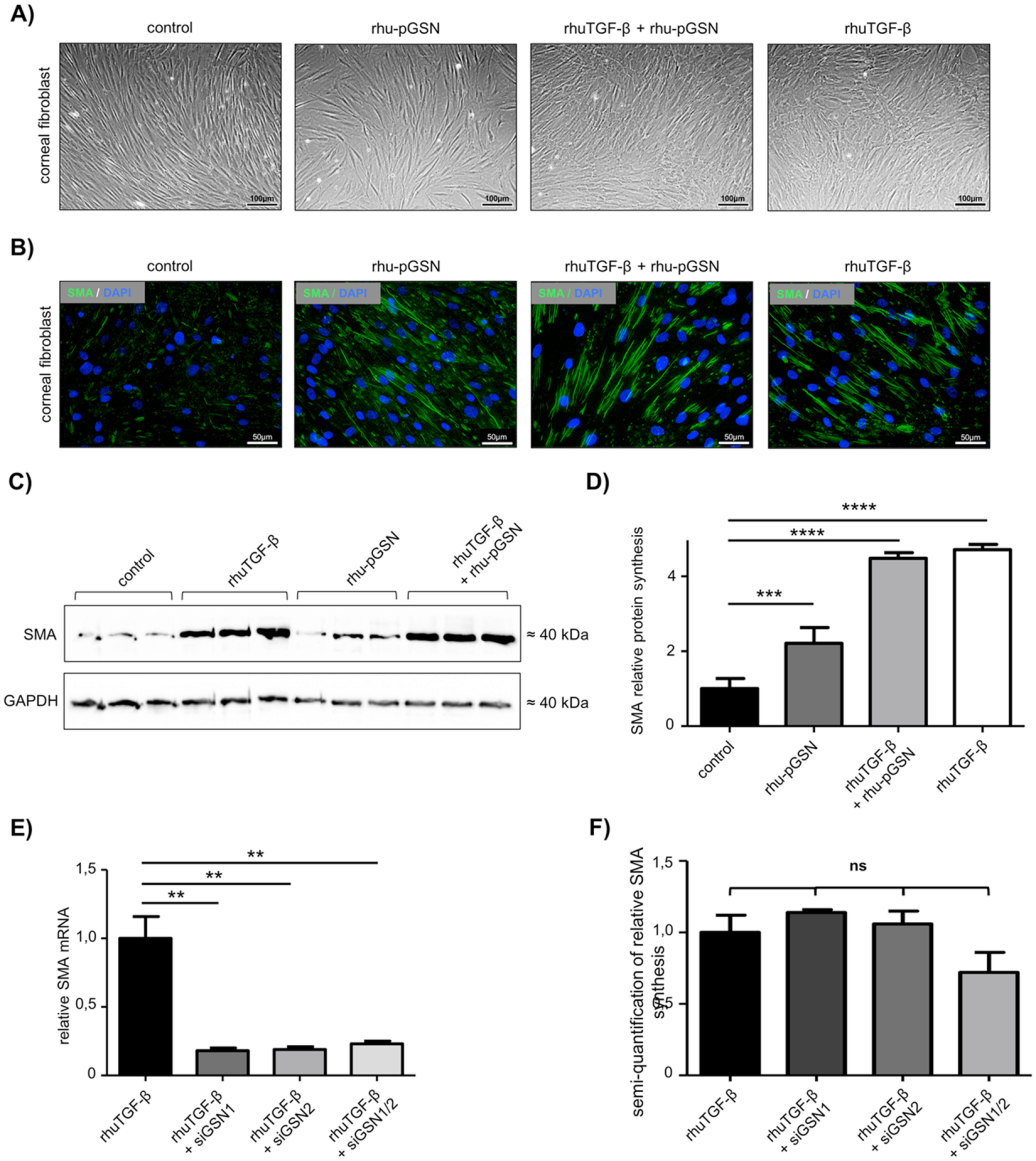
GSN dependent differentiation. (**A**) Representative pictures of cultivated human corneal fibroblasts without stimulation (=control) and after stimulation with rhuTGF-β (3 ng/ml), rhu-pGSN (300 µg/ml) or rhuTGF-β+rhu-pGSN (3 ng/ml + 300 µg/ml) in combination. (**B**) Immunofluorescence of SMA in non-stimulated (=control) and stimulated (either 3 ng/ml rhuTGF-β or 300 µg/ml rhu-pGSN or both in combination) human primary corneal fibroblasts. Unstimulated fibroblasts (=control) only reveal weak SMA reactivity. (**C**) Western blot analysis of unstimulated (=control) and stimulated corneal fibroblasts after stimulation with rhuTGF-β (3 ng/ml), rhu-pGSN (300 µg/ml), or rhuTGF-β + rhu-pGSN (3 ng/ml + 300 µg/ml) using anti-SMA antibody. All samples show a clear band at approx. 40 kDa. GAPDH served as loading control. (**D**) Semi-quantification of relative SMA synthesis from Fig. 4C. Columns are normalized to not stimulated fibroblasts (=control). (**E**) Results of realtime-PCR analysis of SMA-mRNA expression after gene knockdown with siRNA for GSN. The columns are normalized to measured value of group without GSN-siRNA and treated with 3 ng/ml rhuTGF-β. (**F**) Results of semi-quantification of relative SMA synthesis in Western blot analysis (data not shown) after GSN-siRNA gene knockdown. Column is normalized to measured values of human primary fibroblasts treated only with rhuTGF-β. Statistical significance: ns = not significant. Statistical significance: **p < 0.01,***p < 0.005, ****p < 0.0001.

To determine morphological effects of rhu-pGSN and/or recombinant human TGF-β (rhuTGF-β) stimulation, we analyzed human primary corneal fibroblasts stimulated with either one substance or both substances. Control fibroblasts appeared longer, thinner and showed no intracellular granulation compared to stimulated fibroblasts (Fig. 4A). Immunofluorescence revealed subjectively more immunoreactivity to smooth muscle actin (SMA) (green staining) in fibroblasts stimulated with 3 ng/ml rhuTGF-β and 300 µg/ml rhu-pGSN compared to unstimulated corneal fibroblasts (Fig. 4B).

Analysis of SMA by Western blotting after protein isolation detected a singular band at the expected molecular weight (about 40 kDa) in all analyzed samples of stimulated (3 ng/ml rhuTGF-β or 300 µg/ml rhu-pGSN or both in combination) and unstimulated human primary corneal fibroblasts (Fig. 4C). Semi-quantitative densitometry of Western blot analysis revealed significantly (p < 0.0001) higher synthesis of SMA in cells cultured with 300 µg/ml rhu-pGSN or a combination of 3 ng/ml rhuTGF-β and 300 µg/ml rhu-pGSN, each in comparison to control (Fig. 4D). The latter had comparable effects to 3 ng/ml rhuTGF-β alone.

### Amount of smooth muscle actin mRNA is decreased after GSN gene knockdown of both isoforms

Values were normalized for fibroblasts that were treated with 3 ng/ml rhuTGF-β (control). The amount of SMA mRNA was 75% lower in cultivated human corneal fibroblasts treated with 50 nmol GSN-siRNA compared to control (Fig. 4E). This decrease was statistically significant (p < 0.01). In contrast, at the protein level, semi-quantitative densitometry of Western blot analyses (data not shown) after gene knockdown did not show a significant change in SMA synthesis (Fig. 4F).

## Discussion

The goals of our present work were to study the expression patterns of pGSN in tissues from either human or mice ocular surface, to quantify pGSN levels in tear fluid of healthy human subjects and dry eye patients and to investigate the significance of pGSN for corneal wound healing.

Protein size variations for GSN were also described in other journals^28^. The protein band at 80 kDa is specific for the plasmatic form of GSN^18^. The 47 kDa band derives from split of Gelsolin through caspase-3^29^.Interestingly, all cell types related to the ocular surface or participating in tear film production or transport revealed expression and production of pGSN, i.e. corneal and conjunctival epithelial cells (with the exception of goblet cells), acinar epithelial cells of the lacrimal gland and meibocytes as well as epithelial cells of the nasolacrimal duct. This implies multiple origins for the high pGSN concentrations found in tear fluid.

Unexpectedly, the concentrations of pGSN in ocular surface tissues were markedly higher compared to lung, stomach or liver samples, which we used as positive controls. As reported previously, the main sources of pGSN in various vertebrate species are skeletal muscle and smooth muscle myocytes^30^. Abundance of pGSN in the tissues belonging to the ocular surface in comparison to relatively low levels of pGSN in parenchymal organs therefore might indicate an important function of pGSN at the ocular surface and lacrimal apparatus.

To further elucidate this functional significance, samples of human tear fluid from healthy subjects and patients suffering from different forms of dry eye disease (DED) were analyzed regarding their pGSN concentration. Interestingly, pGSN levels of physiological tear fluid were 17-34-fold lower than the aforementioned tissues of the ocular surface. However, tear samples from DED patients revealed a significantly increased concentration of pGSN in comparison to tear fluid from healthy volunteers, while patients with the aqueous-deficient form of DED (ADDE) in particular revealed highly significantly increased pGSN concentrations.

DED is generally characterized by varying amounts of inflammation and tissue damage^31^. Both events are associated with the release of actin and other intracellular components, for example Galectin-3, into the extracellular space and hence into the tear film^32,33^.

Actin, being the most abundant intracellular protein in mammalian cells, has vital roles in cellular integrity, structure and motility, but becomes toxic and develops serious detrimental effects if liberated into the extracellular space secondarily to cell damage^32^.

pGSN is a major player of the extracellular actin-scavenger system, being responsible for clearing free actin from the circulation via the liver and preventing systemic actin toxicity^32^. It also has been shown to be of great importance in elimination of bacterial toxins and regulation of possibly harmful immune responses^23,34,35^. The markedly alleviated pGSN concentration in the tear fluid of DED patients could, hypothetically, be a direct response to an abundance of free actin in DED. In this context it seems that pGSN plays a more important role in the aqueous form of DED than in the evaporative form. This subset of patients is more prone to tear hyperosmolarity, a key driver in ocular surface inflammation, already in the earlier stages of the disease^36^. Hyperosmolarity triggers the release of matrix metalloproteinases and inflammatory cytokines like IL-1β and TNFα, subsequently causing apoptotic death of ocular surface epithelial cells^37^. Further experiments are needed to elucidate whether this is the mechanism of action behind the markedly elevated pGSN concentration in DED, especially ADDE.

pGSN significantly increased the proliferation rate of human corneal epithelial cells *in vitro* (FACS and ECIS®). These findings are consistent with the results of Zhang *et al*., who were able to demonstrate that pGSN has a proliferation-stimulating effect on mesangial cells via the TGF-ß1 pathway^38,39^.

The ocular surface responds to inflammatory events such as bacterial, viral or fungal infections or DED with an upregulation of inflammatory mediators (e.g. IL-1β and TNFα)^40^. As expected, the proliferative effect of rhu-pGSN was partly reduced. Such antiproliferative effects have been well documented for both LPS and TNFα^40,41^. LPS in particular has been reported to decrease GSN levels actively^42^. However, HCE cells treated with rhu-pGSN instead of BSA retained higher proliferation rates even under LPS or TNFα stimulation. This finding is consistent with reports from Cheng *et al*., who identified the ability of recombinant GSN to diminish LPS effects by decreasing systemic TNFα levels as well as by other means^42^.

To investigate further whether rhu-pGSN has the potential to promote wound healing at the ocular surface, the human HCE cell line was studied *in vitro*, and mice bulbs were studied *in vivo/ex vivo*. Our results revealed that a concentration of 300 µg/ml rhu-pGSN significantly increased wound healing in both models. rhu-pGSN has been connected to beneficial effects in various disease models and hypogelsolinaemia, a condition describing lack of pGSN in the circulation, has been associated with poor outcome in numerous conditions^43,44^. However, the value of pGSN for wound healing has not yet been investigated in detail. In this context, our findings are consistent with a report by Rodriguez *et al*., who first described positive immunoreactivity for gelsolin in a case of corneal wound healing in the context of epikeratoplasty^45^. As the corneal stroma is not affected by the “defect inducing procedure” no fibrotic effects result or were observed during or after bathing the eyeballs in rhu-pGSN containing medium.

Finally, the potential of pGSN to induce differentiation of fibroblasts into myofibroblasts was tested *in vitro*. These experiments with fibroblasts were performed to demonstrated, that gelsolin is not only acting on epithelial cells, but also can have effects in deeper corneal layers such as the stroma. In the early phase of wound healing, differentiation of residential fibroblasts into myofibroblasts via TGF-β is an important step in wound closure and a prerequisite for definite healing^46^. It’s know that TGF-β regulates GSN expression^17^. Our study revealed that rhu-pGSN significantly increased the expression of SMA in cultured fibroblasts, which is a morphological marker for differentiation into myofibroblasts^47^. In line with this, gene knockdown of GSN demonstrated a decreased expression of SMA mRNA. These findings indicate that pGSN not only promotes wound healing through TGF-β-associated cascades by affecting epithelial cells, but also by fibroblast transformation. In contrast to the measured SMA mRNA regulation the SMA protein level was not changed. The presumable reason for this is the short incubation time with siRNA for only 24 hours. Our results in human corneal fibroblasts indicate that SMA is increased after treatment with rhu-pGSN suggesting that the regulation of the TGF-β cascade could promote scar formation. Further experiments are needed to elucidate this effect further.

In conclusion, our study demonstrates that pGSN is an important molecular player at the ocular surface, as it is present in the tear film of healthy subjects (at least human and mice) and increased in the tear film of patients suffering from DED. Considering the well-known functions of pGSN in the circulation, a significant role for the regulation of inflammatory conditions of the ocular surface, besides its importance as actin-scavenger, may be postulated. Furthermore, our study shows the beneficial effects of rhu-pGSN for corneal wound healing. These findings may be extrapolated to the wound healing process in general, but further evidence in other, exemplary skin models of wound healing is necessary to confirm our results. Our study identifies rhu-pGSN as a promising target for new pharmacological approaches, aiming to enhance impaired or delayed wound healing in difficult clinical scenarios like diabetes or burn patients.

## Methods Summary

All tissue samples were obtained from cadavers donated to the Department of Anatomy, Friedrich-Alexander-University Erlangen-Nürnberg, Germany. All animals used in this study were treated in accordance with the Association for Research in Vision and Ophthalmology, Resolution on Use of Animals in Ophthalmic and Vision Research and the recommendations of the National Institute of Health Guide for the Care and Use of Laboratory Animals. RNA preparation, cDNA synthesis as well as RT-PCR analysis were performed using the respective primers (Table 3S) as previously described by Schicht *et al*. 2014 (cf. also supplemental part)^48^. Quantitative real-time RT-PCR using cultivated as well as stimulated ocular surface cell lines, i.e. a human corneal epithelial cell line (HCE), a human conjunctival epithelial cell line (HCjE) and a human meibomian gland epithelial cell line (HMGEC), was performed to quantify the respective genes as previously described by Hampel *et al*. 2013 (cf. also supplemental part)^49^. Western blot, ELISA and immunohistochemical localization of the protein was performed as described previously (cf. also supplemental part)^48^. Immunofluorescence analysis and investigation of proliferation enhancing properties using fluorescence-activated cell sorting (FACS) with a bromodeoxyuridine (BrdU) assay were performed as described in Mauris *et al*. 2015 (cf. also in supplemental part)^50^. Electric Cell-Substrate Impedance Sensing (ECIS®) for cell proliferation analysis was performed as described by Hampel *et al*. 2015 (cf. also supplemental part)^51^. A migration assay (scratch assay) with HCE cells was performed as described in Dieckow *et al*. 2016 (cf. also in supplemental part)^52^. The corneal wound healing mouse model has been previously described (cf. also supplemental part)^49,53^. Measurement of smooth muscle actin expression depending on interaction between GSN and TGF-β was performed by gene knockdown. Extraction and cultivation of human primary corneal fibroblasts was performed as described by Hampel *et al*.^49,54^. Stimulation and gene knockdown with human primary corneal fibroblasts is described in the supplemental part.

## Electronic supplementary material

Material and Methods
