# Supplementary material for: Plasma gelsolin promotes re-epithelialization
Source: Sci Rep. 2018 Sep 3;8:13140. doi: 10.1038/s41598-018-31441-2 (PMC6120956; doi:10.1038/s41598-018-31441-2)
Supplement: Supplementary file 1 — Material and Methods [file 41598_2018_31441_MOESM1_ESM.pdf]

Plasma gelsolin promotes re-epithelialization

Wittmann J, Dieckow J, Schroeder H, Hampel U, Garreis F, Jacobi C, Milczarek A, Hsieh KL, Pulli B, Chen JW, Hoogeboom S, Bräuer L, Paulsen FP, Schob S, Schicht M

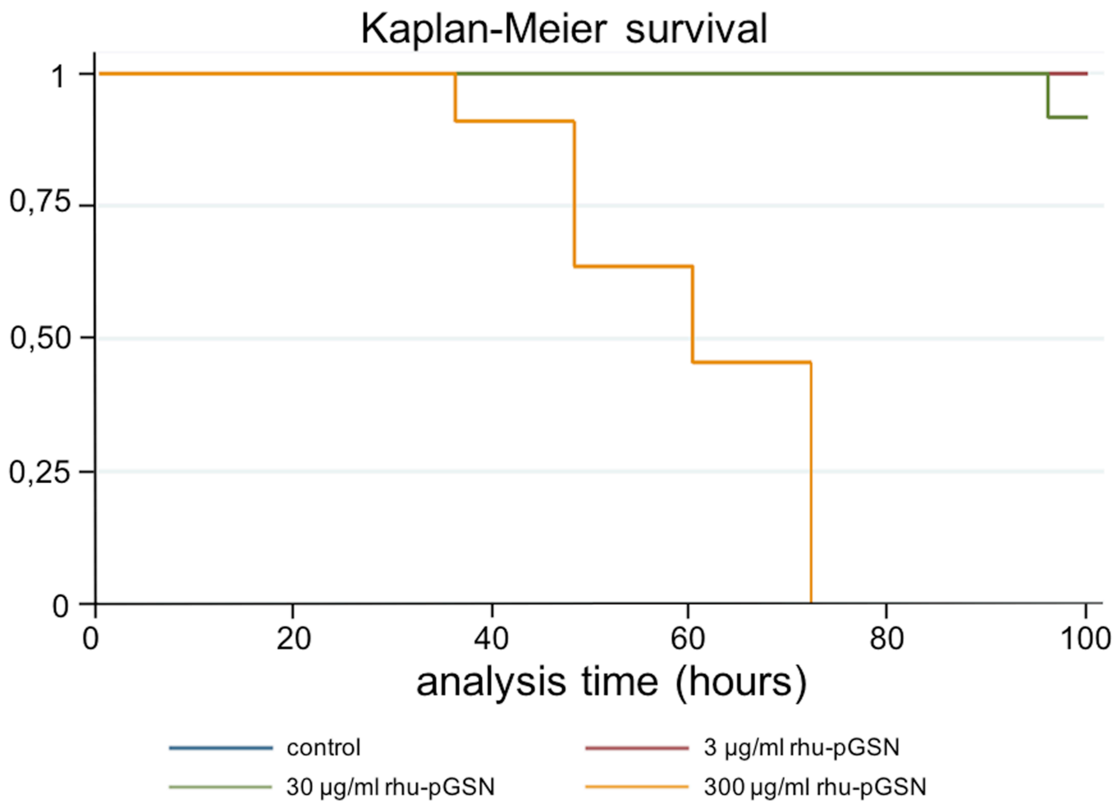

Figure S1: Kaplan-Meier curves show probability of corneal wound healing at different times in placebo (control), 3 µg/ml, 30 µg/ml and 300 µg/ml rhu-pGSN groups. Corneal re-epithelialization was significantly enhanced by local application of 300 µg/ml rhu-pGSN to the wounded corneal surface.

Table S1: Results of ELISA Analysis

**Tissue (human)**	**Mean (ng/mg)**	**Standard error of the mean (SEM) (ng/mg)**	**exp/biol n=**
**Cornea**	750.34	146.15	6
**Conjunctiva**	1110.50	139.52	6
**lacrimal gland**	2011.46	768.30	6
**efferent tear duct**	1646.52	629.85	6
**Eyelid**	1137.12	122.53	6
**Lung**	547,90	163.54	6
**Liver**	156.18	11.95	2
**Stomach**	377.38	15.41	2
**tear fluid control**	64.4	5.70	10 (biol)
**tear fluid ADDE**	926.82	209.46	14 (biol)
**tear fluid EDE**	207.77	55.53	14 (biol)
**Tissue (mouse)**	**Mean** (ng/mg)	**Standard error of the mean (SEM)** (ng/mg)	**experimental n=**
**Cornea**	4.99	0.40	12
**Conjunctiva**	6.93	0.57	12
**lacrimal gland**	3.07	0.27	12
**Eyelid**	5.49	0.39	12
**Lung**	0.39	0.05	10
**Liver**	0.34	0.05	6
**Stomach**	1.24	0.40	10
**tear fluid**	0.09	0.03	3 (biol)

exp = experimental; biol = biological; ADDE = aqueous-deficient dry eye; EDE = evaporative dry eye

Table S2: Antibodies used for protein detection

Primary-Antibody	Dilution	Application	Order number	Company
**Rabbit anti-gelsolin**	tissue 1:200<br>cells 1:500	IHC; WB	sc-48769	Santa Cruz Biotechnology
**Mouse anti-gelsolin**	1:500	IF	GS-2C4	Thermo Fisher Scientific
**Mouse anti-β-actin**	1:1000	WB	sc-47778	Santa Cruz Biotechnology
**Rabbit anti-β-actin**	1:1000	WB	4691S	Cell Signaling Technology
**Mouse anti-SMA**	1:1000	IHC; IF; WB	SMA-L-CE	Leica
**Mouse anti-GAPDH**	1:1000	WB	sc-48166	Santa Cruz Biotechnology
**Secondary-Antibody**				
**Goat anti-mouse (IgG-HRP)**	1:2000 - 1:5000	WB	sc-2005	Santa Cruz Biotechnology
**Goat anti-rabbit (IgG-HRP)**	1:2000 - 1:5000	WB	PO448	Dako Glostrup
**Goat anti-rabbit (IgG-HRP)**	1:2000 - 1:5000	WB	PO448	Dako Glostrup
**Goat anti-mouse (IgG-Alexa 488)**	1:2000 - 1:5000	IF	A-11001	Thermo Fischer Scientific

IHC = immunohistochemistry; IF = immunofluorescence; WB = Western blot

Table S3: Sequences of the primers used for RT-PCR

Primer	Sense	Antisense	pb	Temp.
**Hu GSN**	TGC AGC TGG ATG ACT ACC TG	GAA GCT CTC CCA GGA CAC AG	233 bp	60°C
**Mo GSN**	CCT GTG TCC TGG GAC AGT TT	CTC GGT ACC TTC AGG CAG AG	246 bp	60°C
**Hu β-actin**	GAT CCT CAC CGA GCG CGG CTA CA	GCG GAT GTC CAC GTC ACA CTT CA	298 bp	60°C
**Mo β-actin**	ATA TCG CTG CGC TGG TCG TC	AGG ATG GCG TGA GGG AGA GC	516 bp	60°C
**Hu real GSN**	CCT ATC CCC GGG ACT AAG AC	AGG CAG TGC TGT CCT CTT CT	74 bp	60°C
**Hu real Actin**	AGC GAG CAT CCC CCA AAG TT	GGG CAC GAA GGC TCA TCA TT	285 bp	60°C

Hu = human; Mo =mouse

Supplements Methods

Human tissue

All human tissues were obtained from cadavers donated to Anatomy Department II in Erlangen. For this study only cadavers with no ophthalmic surface disease were used. Tissues were prepared, immediately frozen in liquid nitrogen and stored at -80 °C.
Immunofluorescence experiments using human specimens conducted at the Schepens Eye Research Institute were approved by the Schepens Eye Research Institute Institutional Review Board (IRB#2011-027). These studies were performed using discarded and deidentified human tissue and were deemed to have IRB exempt status not requiring informed consent.

Experimental animals and tissue

All mice used for this paper were C57Bl/6 (Harlan, Harlan Laboratories Inc., Indianapolis, USA). They were treated in accordance with the Association for Research in Vision and Ophthalmology Resolution on the use of Animals in Ophthalmic and Vision Research and the recommendations of the National Institute of Health Guide for the Care and Use of Laboratory Animals. The mice lived under standard laboratory conditions with a 12h light-dark rhythm.
If necessary, animals were killed with volatile anesthetic isoflurane (Abott GmbH & Co KG, Wiesbaden, Germany) and cervical dislocation.
Mouse cadavers (n=6) (C57Bl/6) were used to obtain organs and tissues. They were prepared, immediately frozen in liquid nitrogen and stored at -80°C.

Human Tear fluid

Tear fluid was obtained and treated as described in former studies of the working group[1].

For Western blot analysis human tear fluid of a healthy female and a healthy male donor (age 25-30 years, each n=1) was used. Tear fluid was obtained under same conditions as described below.

Tear fluid samples from healthy volunteers (n=10) and dry eye patients (mean age 55 ± 25 years; mixed gender, each n=14) were collected at the Department of Ophthalmology, Friedrich-Alexander-University Erlangen-Nürnberg, Germany. These specimens were obtained in compliance with good clinical practice and with informed consent **(54-2532.1-35/13)**. Ethical approval was obtained from the Ethics Committee of the University of Erlangen-Nürnberg. Written consent was obtained from all patients and subjects after explanation of the procedures and study requirements. Tears from 10 control subjects were included in the study. They had no symptoms for dry eye disease or ocular discomfort, did not use any artificial tears or lubricant eye drops and did not suffer from any autoimmune disorders or other eye diseases including ocular allergies, and had no history of eye surgery or contact lens wearing. 28 eyes of 28 patients with moderate to severe dry eye (DEWS dry eye severity level 2) were enrolled in the study [2]. Inclusion criteria were typical symptoms measured by a validated questionnaire (particularly descriptions see below): Ocular Surface Disease Index© questionnaire Score (OSDI Score) > 40, a tear break-up time (TBUT) ≤ 10 s, a Schirmer test with anesthesia ≤ 10 mm, and lid-parallel conjunctival folds (LIPCOF) > 2. Patients were divided into two subgroups: evaporative dry eye (EDE, n=14) and aqueous-deficient dry eye (ADDE, n=14). EDE patients had a tear break-up time of ≤ 5 s and a Schirmer test ≤ 10 mm. ADDE patients showed a tear break-up time of ≤ 10 s and a Schirmer test ≤ 5 mm. Exclusion criteria consisted of a medical history of trauma or infection, ocular allergies, pregnancy, lactation, history of refractive surgery / ocular surgery / any other surgery within the previous 6 months, immunosuppressive medications, or the use of contact lenses within 14 days prior to ophthalmological

examination. Also excluded from this study were patients wearing punctum plugs, patients with history or evidence of epithelial keratitis from herpes simplex, patients with recent varicella, corneal or conjunctival viral disease, acute corneal, conjunctival, or palpebral bacterial infection or ocular fungal infection. The following test measurements were accomplished with all patients.

The OSDI is a subjective symptom questionnaire that measures the severity of dry eye disease. It includes 12 items regarding visual function, ocular symptoms, and environmental triggers queried for the past week. Possible answers for the questions are as follows: none of the time, some of the time, half of the time, most of the time and all of the time (0-4). The published scoring algorithm contains scores from 0 to 100 [3].The OSDI is used as a measure of outcome in randomized controlled trials [4].

LIPCOF (degree 0-3) were evaluated by slit-lamp examination. The classification of LIPCOF according to Höh et al. was used [5]. With degree 0, no permanent conjunctival fold exists. Degree 1 describes the permanent presence of an individual fold, which does not exceed the height of the normal tear meniscus. At degree 2, the LIPCOF disintegrates into 2 or several small parallel folds that are lower than the normal tear meniscus. If there are several parallel conjunctival folds exceeding the height of the normal tear meniscus, degree 3 applies.

For diagnosing tear film stability, a standardized measurement was taken of tear film break-up time in seconds. 5 µl of non-preserved 2% sodium fluorescein were instilled onto the bulbar conjunctiva using a micropipette without inducing reflex tearing. Then the patient was instructed to blink normally without squeezing several times to distribute the fluorescein, then refrain from blinking until told otherwise. The slit lamp magnification was set at 10x and a Wratten 12 yellow filter was used to enhance observation of the tear film over the entire cornea [6].A stopwatch was used to record time between last complete blink and the first disruption of the tear film. After observing

the TBUT, the patient was instructed to blink normally again. Three measurements were taken as recommended by the DEWS report 2007, and the average was calculated [7].

The Schirmer test was performed for classification of DED and extraction of tear fluid. After topical anesthesia with one drop oxybuprocaine-HCL (Conjuncain-EDO®), a Schirmer test strip (35 x 5 mm; Schirmer Tear Test, Ophthalmic Strips Mark Blu, Optitech Eyecare Europe BVBA, Gingelom (Kortijs), Belgium) was placed in the lower outer fornix, after which the patient was instructed to close his/her eyes. After 5 minutes, the strip was removed from the eye and the length of wetting was measured[4].In addition, the tear fluid-soaked Schirmer strip was transferred to a 1.5 ml reaction tube and stored at -80˚C for further testing.

Isolation of the tear fluid from Schirmer strips

Retrieving the fluid from the strip (n=38) was done similarly to the description by Posa et al. [8]. For extraction of the tear fluid, the Schirmer strips were transferred to an 0.5 ml tube punctured at the bottom with a cannula. The tube was placed in a larger (1.5 ml) tube and centrifuged at 14000 rpm for 5 minutes. The centrifugal force pulled the tear fluid out of the Schirmer strip, through the central “pore” in the bottom of the smaller tube and into the outer 1.5 ml tube [8]. After centrifugation the amount of tear fluid obtained was measured, and the difference to 60 µl was added by pipetting aqua ad injectabilia (Braun, Melsungen, Germany) onto the strip and centrifuging again at 12300 rpm for 2 minutes (Scan Speed mini, Labogene, Lynge, Denmark). The tear fluid, thus prepared, was stored at -20°C.

Mouse tear fluid

Mouse tear fluid was obtained from 3 mice. To dissolve proteins of mouse tear film, 1.5 µl phosphate buffered saline (PBS) was applied to each eye. Afterwards, fluid was obtained with a 2 µl capillary (Sigma-Aldrich, Munich, Germany) from the lateral corner of the eye. The obtained fluid was pooled and stored at -20°C.

To obtain the volume required for ELISA, the mouse tear fluid obtained was topped up to 50 µl with the standard diluent of the ELISA kit.

Human cell lines

All cells were cultured in an incubator under standard conditions (37°C, 5% CO_2, 21% O_2).

SV 40-transformed human cornea epithelial cells (HCE) (passage 62; obtained from Kaoru Araki-Sasaki, Tane Memorial Eye Hospital, Osaka, Japan) were cultured in Dulbecco's modified Eagle's medium HAMS F12 (DMEM F-12 HAMS) (Biochrome AG, Berlin, Gemany) with additional 10% fetal bovine serum (Thermo Fisher Scientific, Waltham, USA). Human conjunctiva epithelial cells (HCjE) (passage 24; obtained from Yolanda Diebold, University Institute of Applied Ophthalmobiology [IOBA], University of Valladolid, Valladolid, Spain) were kept in DMEM HAMS F12 medium with additional bovine pituitary extract, epidermal growth factor (Sigma Aldrich, St. Louis, Missouri, USA), calcium chloride and penicillin/streptomycin.

Human meibomian gland epithelial cells (HMGEC) (passage 39; provided by David Sullivan (Schepens Eye Research Institute) were cultured as described in earlier paper by Schröder et al. [9]. In brief, HMGEC were cultured in serum-free medium (keratinocyte serum-free medium containing 50 µg/ml bovine pituitary extract and 5 ng/ml epidermal growth factor). These cells are designated HMGEC SFM. After reaching a confluence at about 80 - 90%, serum-free medium was changed to serum-containing medium (DMEM F-12 HAMS containing 10% fetal calf serum + 10 ng/ml epidermal growth

factor) to induce differentiation of HMGEC. From this point on, the cells are named HMGEC SCM.

Human primary fibroblasts were obtained from donated corneas (n=3). We used the remains from transplanted corneas. The corneal stroma was cut into pieces, placed on a petri dish and cultivated with DMEM F-12 HAMS medium (Biochrome AG, Berlin, Gemany) containing 10% FKS (Thermo Fisher Scientific, Waltham, USA). Medium was changed at least every second day. To ensure use of primary fibroblasts only, cells were used only after four subsequent passages.

Isolation of RNA (tissues and cells)

For isolation of RNA human tissues: cornea (n=3), conjunctiva (n=3), lacrimal gland (n=3), efferent tear duct (n=3), eyelid (n=3), lung (n=3), liver (n=1) and stomach (n=1) was used. Mouse tissues were: cornea (n=6), conjunctiva (n=6), lacrimal gland (n=6), eyelid (n=6), lung (n=5), liver (n=6) and stomach (n=5). Human cell lines were: HCE, HCjE, HMGEC SFM, HMGEC SCM (each n=3).

RNA was isolated by TriFast^{TM} (peqGold, peqlab, Erlangen, Germany) and chloroform (Carl Roth GmbH + Co. KG, Karlsruhe, Germany). Precipitation of DNA was done by use of isopropyl alcohol. Pellet was dissolved in 50 µl of DEPC-water. Digestion of DNA was performed by adding a mix of 2 µl DNAse I (RNAse free, 1 u/µl, Thermo Fisher Scientific, Waltham, USA), 6 µl 10x buffer (reaction buffer with $MgCl_2$ for DNAse I, Thermo Fisher Scientific, Waltham, USA) and 2 µl Ribolock RNase Inhibitor (40 u/µl, Thermo Fisher Scientific, Waltham, USA). Reaction was stopped with EDTA 50 mM (Thermo Fisher Scientific, Waltham, USA). Precipitation of RNA was done with 3 M Na-acetate and isopropyl alcohol. Washing steps were done with 75% ethanol in DEPC.

Concentration of RNA in the samples was measured using NanoDrop™ (NanoDrop 2000c Spectrophotometer, Thermo Fisher Scientific, Waltham, USA).

Synthesis of cDNA

For cDNA-synthesis 2 µg mRNA was used. 1 µl Oligo $(dT)_{18}$Primer (Thermo Fisher Scientific, Waltham, USA) was added and the whole solution was incubated at 65°C for 5 minutes. Afterwards 7 µl mastermix (Containing 4 µl 5x reactionbuffer (Thermo Fisher Scientific, Waltham, USA), 2 µl dNTP Mix (Thermo Fisher Scientific, Waltham, USA), 0.5 µl RNase Inhibitor (RiboLock RNase Inhibitor, 40 u/µl, Thermo Fisher Scientific, Waltham, USA) and 0.5 µl reverse transcriptase (Revert Aid H Minus reverse Transcriptase, Thermo Fisher Scientific, Waltham, USA) was placed in each cup. Solution stayed at 42°C for 1 hour and was then stored at -20°C.

Polymerase Chain Reaction

A mix of 2 µl 10x PCR Reaction buffer (without $MgCl_2$) (Invitrogen Thermo Fisher Scientific, Waltham, USA), 2 µl $MgCl_2$ (50 mM) (Thermo Fisher Scientific, Waltham, USA), 2 µl dNTP-mix (10 mM) (Thermo Fisher Scientific, Waltham, USA), 1 µl forward primer (Tab. 1), 1 µl reverse primer (Tab. 1) and 0.2 µl Taq-polymerase (5 U/µl, Thermo Fisher Scientific, Waltham, USA) was used, as well as 9.8 µl of DEPC-H_2O for GSN or 10.8 µl DEPC-H_2O for beta-actin. 2 µl cDNA templates were used for GSN and 1 µl for actin.

Program for GSN was: 95°C 5 minutes, 36 cycles of 30 s 95°C, 30 s 60°C, 40 s 72°C.

For β-actin: 5 minutes 94°C, 30 cycles of 30 s 94°C, 20 s 60°C, 30 s 72°C.

All steps were repeated with a cycled template.

After cycling 5 µl 6x Orange DNA Loading Dye (#RO 631, Thermo Fisher Scientific, Waltham, USA) was added to each tube. Afterwards samples were transferred to a

1.5% agarose gel (Sigma, Merck, Darmstadt, Germany). After gel electrophoresis, pictures were taken with Dark Hood DH-50 and Gerix 1000 (Biostep GmbH, Jahnsdorf, Germany).

Real-time PCR

Gene expression was analyzed by quantitative Real-Time RT-PCR (qPCR) using a LightCyler480® system (Roche, Basel, Switzerland). The PCR reaction contained 10 µl SYBR™ Green mastermix, 0.25 µl of each primer (Tab. 1) and 2 µl of each cDNA, and 7.5 µl nuclease-free water. In each 96-well plate qPCR was performed with a cycle of 5 min 95°C, 55 cycles at 15 s 95°C, 30 s 60°C and 1 s 72°C, to confirm amplification of specific transcripts. A standard curve was generated by serial dilutions of cDNA from non-stimulated cells. To standardize mRNA concentration, the transcript levels of the housekeeping gene β-actin (ACTB) were determined in parallel for each sample, and relative transcript levels were corrected by normalization based on the ACTB mRNA transcript levels. All qPCRs were performed in triplicate, and the changes in gene expression were calculated by the ΔΔCt method.

Isolation of proteins (tissue and cells)

Frozen tissue (human tissues: cornea (n=3), conjunctiva (n=3), lacrimal gland (n=3), efferent tear duct (n=3), eyelid (n=3), lung (n=3), liver (n=1), stomach (n=1); Mouse tissues: cornea (n=6), conjunctiva (n=6), lacrimal gland (n=6), eyelid (n=6), lung (n=5), liver (n=6) and stomach (n=5); human cell lines: HCE, HCjE HMGEC SFM and HMGEC SCM (each n=3)) as obtained above, was placed in a lysis tube (innuSpeed Lysis tube A, Analytik Jena, Biometra, Jena, Germany). 300 µl of master mix (1 ml 1% triton-X-100-buffer (Roth, Karlsruhe, Germany) containing 10 µl protease inhibitor and 10 µl phosphatase inhibitor was added. It was crushed in a speed mill (Speed Mill plus,

analytik Jena, Jena, Germany) for 2 minutes and incubated on ice for 30 minutes, then centrifuged 40 minutes at 4°C and 13000 rpm. Supernatant was transferred into a new cup, protein concentration was measured and the sample stored at -80°C.

Western blot analysis

30 µg protein was given to every lane of a 12% SDS-polyacrylamide gel. 5 µl Page Ruler™ Prestained Protein Ladder (product#26616, Thermo Fisher Scientific, Waltham, USA) was used as marker. Electrophoresis run 30 minutes in stacking gel at 10 mA per gel and then 20 mA per gel in resolving gel. Proteins were plotted to nitrocellulose blotting membrane (Amersham™ Hybond ECL, GE Healthcare, Chalfont St. Gilles, UK) with 300 volts, 40 mA for 2 hours. To confirm whether proteins were on the membrane, ponceau red (Sigma Aldrich, St. Louis, Missouri, USA) was used and rinsed with aqua dest. The membrane was blocked with 5% skim milk powder (Blotting grade, low fat, Roth, Karlsruhe, Germany) in phosphate buffered saline with Tween 20 (PBST) for 30 minutes. Primary antibodies (Tab. S2, available at www.aaojournal.org) were diluted in milk solution and placed on the membrane overnight at 4°C. Secondary antibodies (Tab. S2, available at www.aaojournal.org) were diluted with 5% skim milk powder as above and placed on the membrane for 2 hours at room temperature. After washing with PBST, Chemiluminescent HRP Substrate (1:1 HRP Substrate Peroxide Solution and HRP Substrate Luminol Reagenz, Immobilin™ Western, Millipore, Darmstadt, Germany) was placed on the membrane for 5 minutes. Pictures were made by use of BioRad Universal Hood II (BioRad Laboratories, Hercules, USA). Verification was done by use of antibodies to either beta actin or glycerinaldehyde-3-phosphate dehydrogenase (GAPDH). Stomach, lung and liver were used as positive controls. The anti-GSN antibody used binds to amino acids 596-665 at the C-terminus, which is part of both GSN isoforms. Semi-quantitative statistic analysis of the Western blot analysis

was done by use of Bio Rad Quantity one® analysis software (version 4.6.9., Bio-Rad Laboratories, Hercules, USA).

Histology and immunochemistry

Human and murine tissue (cornea, conjunctiva, meibomian gland, lacrimal gland, stomach and lung (each n=3)) was fixed in 4% paraformaldehyde (PFA) (Roth, Karlsruhe, Germany), dehydrated and embedded in paraffin. Slices were cut with a thickness of 5 µm. After Xylol and decreasing alcohol (100% / 100% / 100% / 96% / 96% / 80% / 70%), hydrogen peroxide (hydrogen peroxid 30%, Rotipuran®, p.a., Carl Roth GmbH, Karlsruhe, Germany) was applied. Then slides were heated for 10 minutes in boiling citrate buffer (pH=6). After cooling down for at least 1 hour, normal goat serum (Tab. S2, available at www.aaojournal.org) was added for 20 minutes. As the blocking kit, Avidin Solution ready to use (Thermo Fisher Scientific, Waltham, USA) and d-Biotin Solution ready to use (Thermo Fisher Scientific, Waltham, USA) were used. The primary antibody to GSN was diluted with TBST 1:50-1:100. It stayed on the slides overnight at 4°C and an additional hour at room temperature. The secondary antibody (dilution 1:200) (Tab. S2, available at www.aaojournal.org) was applied to the slides for 2 hours. ABC-Kit (Vectastain ABC, Elite PK-600 Standard, Vector Laboratories INC., Burlingame, USA) was added for 1 hour. To develop coloration, AEC substrate (AEC + High Sensitivity substrate chromogen ready to use, Dako, North America Inc., Carpinteria, USA) was used. Afterwards slides were stained with hemalum for 40 seconds, rinsed with aqua dest. and covered with Aquatex (Merck, Darmstadt, Germany).

Pictures were taken using the microscope Biorevo BZ-9000 (Keyence Deutschland GmbH, Neu-Isenburg, Germany).

Immunofluorescence histology

Cryosections of human eyelid tissue (n=3) and human lacrimal gland (n=3) in OCT (5 µm thick) were brought to and kept at room temperature for 30 min. In a second step the sections were fixed with ice-cold methanol for 5 minutes and air dried at 37°C for 30 min. Following hydration in phosphate-buffered saline (PBS) for 10 minutes, sections were rinsed in PBS-Tween 20 for 2 x 2 min and subsequently blocked in species-specific blocking solution (2% rabbit serum, 1% BSA, 0.1% Triton X, 0.05% Tween 20, 0.05% sodium azide, 0.1 M PBS pH 7.2) for 30 minutes. After this, sections were incubated with primary anti-gelsolin antibody (Tab. S2, available at www.aaojournal.org) in 3% bovine serum albumin (BSA) in PBS for 1 hour at room temperature in a moist chamber. Incubation with primary antibodies was routinely omitted in control experiments. After rinsing with PBS-Tween 20, 3 times for 2 minutes each, the corresponding secondary antibody (Tab. S2, available at www.aaojournal.org) was applied for 1 hour at room temperature. Slides were then rinsed, mounted in VectaShield mounting medium containing DAPI (Vector Laboratories, Burlingame, USA), and observed under a fluorescence microscope (Nikon Eclipse E-400, Tokyo, Japan).

Enzyme-Linked Immunosorbent Assay

A commercially available Enzyme-Linked Immunosorbent Assay (ELISA) kit (SEA372Mu, SEA372Hu, Cloud-Clone Corp., Houston, USA) was used as described in the manufacturer's protocol. Analyzed tissues were: Human: cornea (n=3), conjunctiva (n=3), lacrimal gland (n=3), efferent tear duct (n=3), eyelid (n=3), lung (n=3), liver (n=1), stomach (n=1); Mouse: cornea (n=6), conjunctiva (n=6), lacrimal gland (n=6), eyelid (n=6), lung (n=5), liver (n=6), stomach (n=5), tear fluid (n=3); human tear fluid: healthy (=control) (n=10), aqueous-deficient dry eye (n=14),

hyperevaporative dry eye (n=14). The antibody in the kit binds amino acid sequence Phe76 to Ala504. This sequence is part of both GSN isoforms. Every probe was applied twice except mouse tear fluid, mouse liver and human tear fluid. The analysis was performed using a microplate spectrophotometer (ELISA reader; vmax. kinetic microplate reader; molecular devices, MWG-Biotech, Ebersberg, Germany) to measure absorbance (450 nm and 405 nm). Concentrations in the samples are expressed in nanograms of gelsolin per milligram of total protein.

Proliferation assay I (BrdU and fluorescence-activated cell sorting (FACS))

Bromodeoxyuridine (BrdU) incorporation and flow cytometry analysis were used to assess cell cycle progression. Telomerase-immortalized human corneal keratinocytes (HCE) were grown as a monolayer in keratinocyte serum-free medium (K-SFM) supplemented with 0.2 ng/ml epidermal growth factor (EGF), 44 mg/ml $CaCl_2$, 25 µg/ml bovine pituitary extract and 1% penicillin/streptomycin (all Life Technologies, Carlsbad, USA). Before confluence was achieved, cells were incubated for 12 h with 1 µg/ml LPS (lipopolysaccharide) (n=3) or 10 ng TNFα (both Merck, Darmstadt, Germany) (n=3), respectively, followed by staining using the APC BrdU Flow Kit (BD Pharmingen, Franklin Lakes, USA) according to the manufacturer's protocol. Briefly, 10 µM BrdU was added to the culture media (with or without supplements mentioned above). Cells were harvested 12 h after BrdU addition, fixed and permeabilized. Incorporated BrdU was exposed by treatment with DNase for 1 h at 37°C. Cells were subsequently stained with APC-anti-BrdU antibody (1:50) and analyzed on a BD LSRII flow cytometer (Becton Dickinson, Franklin Lakes, USA). Cells from the same population (each n=3) that were not exposed to BrdU were used as a negative staining control to determine background- staining levels for the anti-BrdU monoclonal antibody.

Proliferation assay II (ECIS®)

For cell proliferation, immortalized human corneal epithelial cells were used. To measure proliferation, the Electric Cell-Substrate Impedance Sensing (ECIS®) system (Applied BioPhysics Inc., NY, USA) was used. The slides used were ECIS Cultureware™ 8W10E+PET (Applied BioPhysics Inc., NY, USA). Setting was as stated in the manufacturer's protocol. In brief, slides were prepared with 400 µl normal cultivation medium (DMEM/ HAMS F-12 (Biochrome AG, Berlin, Germany) containing 10% fresh bovine serum (Life Technologies Corp., Grand Island, USA) and 10% penicillin/streptomycin (Sigma Aldrich, Munich, Germany) overnight. The fluid was replaced with 400 µl cell dilution (c=2.0 x 10^5 cells/ml) and left overnight. On day three, medium was replaced with 400 µl of stimulation fluid. For stimulation, recombinant human plasmatic gelsolin (rhu-pGSN) (Biogen Inc, Cambridge, USA) was used in concentrations of either 30 µg/ml (n=7) or 300 µg/ml (n=8). BSA was used as a control substance (n=6). Stimulation medium was changed every two days. Cells were cultured under standard conditions (37°C, 5% CO_2, 21% O_2). According to manufacturer's protocol, the measured impedance at 4000 Hz is a mix of cell-coverage and changes in the spaces either under or between the cells. We designate it as a display of cell proliferation. So the rate of proliferation is equal to the measured impedance. For statistical analysis, resistance at 4000 Hz was used. The impedance was normalized to measured impedance values after 24 hours of cultivation prior to stimulation.

Cell culture and wound healing assay (Scratch Assay)

HCE cells were grown to confluence in the above-mentioned medium. Using pipette tips, the cell layer was scratched several times, creating "wounds" of similar width. The cells were washed twice with PBS to remove debris, and fresh medium was applied.

Images of wounded areas were taken and areas were marked for later observation. Cells were subsequently stimulated with 300 µg/ml rhu-pGSN (n=3). The negative control (n=3) contained no rhu-pGSN, while BSA (300 µg/ml) (n=3) served as a protein control. The previously imaged areas were photographed again after 24 hours of stimulation. The wounded area was assessed at 0 hours as well as 24 hours using Adobe Photoshop. Stimulated samples were compared to control values.

Combined *in vivo/ex vivo* model of corneal wound closure

To investigate the biological roles of rhu-pGSN in corneal damage and repair and assess the dose and effects of exogenously administered rhu-pGSN, a combined *in vivo/ex vivo*model of corneal epithelial injury in mice **(animal experiment request number 54-2532.1-35/13)** was used that has been described previously [10,11]. Ethical approval was obtained from the Ethics Committee of the University of Erlangen-Nürnberg. For this, 12-week-old female mice (n=24) were used. They were anesthetized with a mix of Ketavet (ketamine hydrochloride 100 mg/ml, Pfizer, NY, USA) and Xylazine (Rompun 2%, Bayer, Leverkusen, Germany) intraperitoneally according to body weight. Eyes were treated with local anesthetic. Then a corneal defect was induced on both eyes by use of a 2 mm in diameter alkali-soaked (0.5 M NaOH) filter paper disc. It was left on each eye for 2 minutes. Then the eyes were washed with sterile 0.9% NaCl (sodium chloride ≥ 99.5 p.a., ACS, ISO M 58.44G/mol, volumic mass 2.17, Carl Roth GmbH & Co KG, Karlsruhe, Germany). After creation of the corneal defect, the mice lived for 6 hours and were then sacrificed. Eyeballs were enucleated and transferred to a well filled with dental wax. For cultivation serum-free Dulbecco's modified Eagle's medium (DMEM HAMS F-12, Biochrome AG, Berlin, Germany) with 10% penicillin/streptomycin (Sigma Aldrich, Munich, Germany), amphothericine B (250 µg/ml, Biochrome Ag, Berlin, Germany) and rhu-pGSN (rhu-

pGSN BG 9385, Biogen Inc, Cambridge, USA) was used. Concentration of rhu-pGSN was 3 µg/ml, 30 µg/ml or 300 µg/ml (each n=6). As control a placebo (n=6) (Placebo/Diluent for BG 9385, Biogen INC, Cambridge, USA) was used. 1 ml of medium with rhu-pGSN was placed on each eye. The plates were stored in incubator under standard conditions (37°C, 5% CO_2 and 21% O_2). To measure the defect area, medium was removed and 1 µl of fluorescein solution was applied to the eyes. To produce this fluorescein solution, a strip (Fluorescein Paper, Haag-Steit AG, Koeniz, Switzerland) was dissolved in 1.5 ml 0.9% NaCl. Blue light was generated by an endoscopic system from Karl Storz (Tuttlingen, Germany) (endoscope flash generator 600 type 600BA, 0° optic system 7200A, blue filter 20100032, filter 20100033 and light cable 495NB). Photos were taken after 0, 6, 12, 24, 36, 48, 60, 72, 84 and 96 hours with a Canon Eos 700D (Tokyo, Japan) with camera lens from Karl Storz (Germany PV 593 A EO Japan). After taking the pictures, fluorescein was rinsed with warm sterile 0.9% NaCl, after which medium was again added to the enucleated eyes fixed in dental wax.

To evaluate the area of the corneal defect, SigmaScan Pro Image analysis software (Version 5, SPSS Inc, Chicago, USA) was used. The wound surface was measured in proportion to the whole surface of the eye.

Expression of smooth muscle actin in human fibroblasts.

Primary human fibroblasts were directly cultured on object slides. Fibroblasts were stimulated either with recombinant human transforming growth factor beta (rhuTGF-β) (Bio-techne/R&D Systems™, Minneapolis, USA) in a concentration of 3 ng/ml or with rhu-pGSN (rhu-GSN BG 9385, Biogen Inc., Cambridge, USA) in a concentration of 300 µg/ml or with both stimulants in combination (each n=3). Cells were stimulated for 48 hours. Afterwards cells were fixed onto slides by use of ice cold methanol at -20°C,

then washed with PBST followed by hyaluronidase for 30 minutes. Then native pictures and immunofluorescence was performed as described above.

Gen knockdown with siRNA for GSN

For gene knockdown primary corneal fibroblasts and small interfering RNA (siRNA) (each n=3) custom-made by Sigma Aldrich (Darmstadt, Germany) was used. Sequences were siGSN1: sense: 5'-CUGUUGAGGUAUUGCCUAA-3', anti-sense: 5'-UUAGGCAAUACCUCAACAG-3'; siGSN2: sense: 5'-GACUGAAGGCCACACAGGU-3', anti-sense: 5'-ACCUGUGUGGCCUUCAGUC-3'. These sequences were part of both isoforms of GSN. Negative control (n=3) was accomplished by use of MISSION® siRNA Fluorescent Universal Negative Control #1, 6-FAM (Sigma Aldrich, Darmstadt, Germany). According to attached manufacturer’s manual about 45000 cells were applied to each well and cultured for 24 hours until a confluence of about 30-50% was achieved. Mastermix of transfection medium was prepared as follows for each well: 200 µl serum-free DMEM with penicillin/streptomycin, 50 nmol siRNA and 6 µl MISSION® siRNA Transfection Reagent (Sigma Aldrich, Darmstadt, Germany) were mixed and incubated at room temperature for 12 minutes. During this time, medium from fibroblasts was replaced. Prepared mastermix was placed on every dish and left there for 24 hours. Afterwards, cells were cultured with normal-serum free medium for another 24 hours. Finally, cells were stimulated for an additional 24 hours with 3 ng/ml rhuTGF-β (Bio-techne/R&D Systems™, Minneapolis, USA). Fibroblasts without siRNA were run parallel as controls. Afterwards, either proteins or RNA were isolated and analyzed as described above.

Statistical analysis

All statistical analyses were performed using GraphPad 5 (GraphPad Prism version 5.04, GraphPad Software Inc., La Jolla, USA), except for the combined *in vivo/ex vivo* mouse corneal wound healing model. This was done using STATA 12.0 (StataCorp LP, Texas, USA); ELISA: GSN tear fluid: mean ± SEM, one-way variance analysis (ANOVA); ECIS: mean ± SEM, Wilcoxon matched-pairs significance test, paired t-test; scratch assay: mean ± SD, one-way variance analysis (ANOVA); Mouse model: Kaplan-Meier survival estimates; fibroblast stimulation: mean ± SEM, unpaired t-test; fibroblast stimulation with siRNA: mean ± SEM, unpaired t-test.

1 Schicht, M. *et al.* Palate Lung Nasal Clone (PLUNC), a Novel Protein of the Tear Film: Three-Dimensional Structure, Immune Activation, and Involvement in Dry Eye Disease (DED). *Investigative ophthalmology & visual science* **56**, 7312-7323, doi:10.1167/iovs.15-17560 (2015).

2 Craig, J. P. *et al.* TFOS DEWS II Definition and Classification Report. *Ocul Surf* **15**, 276-283, doi:10.1016/j.jtos.2017.05.008 (2017).

3 Schiffman, R. M., Christianson, M. D., Jacobsen, G., Hirsch, J. D. & Reis, B. L. Reliability and validity of the Ocular Surface Disease Index. *Archives of ophthalmology* **118**, 615-621 (2000).

4 Jacobi, C., Jacobi, A., Kruse, F. E. & Cursiefen, C. Tear film osmolarity measurements in dry eye disease using electrical impedance technology. *Cornea* **30**, 1289-1292, doi:10.1097/ICO.0b013e31821de383 (2011).

5 Hoh, H., Schirra, F., Kienecker, C. & Ruprecht, K. W. [Lid-parallel conjunctival folds are a sure diagnostic sign of dry eye]. *Der Ophthalmologe : Zeitschrift der Deutschen Ophthalmologischen Gesellschaft* **92**, 802-808 (1995).

6 Tomlinson, A. & Khanal, S. Assessment of tear film dynamics: quantification approach. *Ocul Surf* **3**, 81-95 (2005).

7 DEWS-Report. The definition and classification of dry eye disease: report of the Definition and Classification Subcommittee of the International Dry Eye WorkShop (2007). *Ocul Surf* **5**, 75-92 (2007).

8 Posa, A. *et al.* Schirmer strip vs. capillary tube method: non-invasive methods of obtaining proteins from tear fluid. *Ann Anat* **195**, 137-142, doi:10.1016/j.aanat.2012.10.001 (2013).

9 Schroder, A. *et al.* In vitro effects of sex hormones in human meibomian gland epithelial cells. *Experimental eye research* **151**, 190-202, doi:10.1016/j.exer.2016.08.009 (2016).

10 Paulsen, F. P. *et al.* Intestinal trefoil factor/TFF3 promotes re-epithelialization of corneal wounds. *J Biol Chem* **283**, 13418-13427, doi:10.1074/jbc.M800177200 (2008).

11 Hampel, U. *et al.* Insulin-like factor 3 promotes wound healing at the ocular surface. *Endocrinology* **154**, 2034-2045, doi:10.1210/en.2012-2201 (2013).

Statistical Supplement for Figure 3 (I)

One-way Analysis of Variance (ANOVA)

The P value is < 0.0001, considered extremely significant.
Variation among column means is significantly greater than expected by chance.

Tukey-Kramer Multiple Comparisons Test
If the value of q is greater than 3.431 then the P value is less than 0.05.

Comparison	Mean Difference	q	P value
Kontrolle vs Gelsolin	0.6345	6.638	*** P<0.001
Kontrolle vs BSA	0.1628	1.662	ns P>0.05
Gelsolin vs BSA	-0.4717	6.121	*** P<0.001

Difference	Mean Difference	95% Confidence Interval From	To
Kontrolle - Gelsolin	0.6345	0.3065	0.9625
Kontrolle - BSA	0.1628	-0.1733	0.4989
Gelsolin - BSA	-0.4717	-0.7361	-0.2073

Assumption test: Are the standard deviations of the groups equal?

ANOVA assumes that the data are sampled from populations with identical SDs. This assumption is tested using the method of Bartlett.

Bartlett statistic (corrected) = 0.000
The P value is > 0.9999.
Bartlett's test suggests that the differences among the SDs is not significant.

Assumption test: Are the data sampled from Gaussian distributions?

ANOVA assumes that the data are sampled from populations that follow Gaussian distributions. This assumption is tested using the method Kolmogorov and Smirnov:

Group	KS	P Value	Passed normality test?
Kontrolle	0.1778	>0.10	Yes
Gelsolin	0.1163	>0.10	Yes
BSA	0.1497	>0.10	Yes

Intermediate calculations. ANOVA table

Source of variation	Degrees of freedom	Sum of squares	Mean square
Treatments (between columns)	2	3.427	1.713
Residuals (within columns)	45	5.180	0.1151
Total	47	8.607	

F = 14.885 =(MStreatment/MSresidual)

Summary of Data

Group	Number of Points	Mean	Standard Deviation	Standard Error of Mean	Median
Kontrolle	9	1.004	0.2929	0.09765	1.095
Gelsolin	21	0.3694	0.2181	0.04760	0.3785
BSA	18	0.8411	0.4565	0.1076	0.7451

Group	Minimum	Maximum	95% Confidence Interval From	95% Confidence Interval To
Kontrolle	0.5522	1.428	0.7787	1.229
Gelsolin	0.01138	0.7412	0.2701	0.4687
BSA	0.05954	1.738	0.6141	1.068

* * *
